# Human haptic perception is interrupted by explorative stops of milliseconds

**DOI:** 10.3389/fpsyg.2014.00292

**Published:** 2014-04-09

**Authors:** Martin Grunwald, Manivannan Muniyandi, Hyun Kim, Jung Kim, Frank Krause, Stephanie Mueller, Mandayam A. Srinivasan

**Affiliations:** ^1^Haptic-Research Laboratory, Paul-Flechsig-Institute for Brain Research, University of LeipzigLeipzig, Germany; ^2^Department of Applied Mechanics, Biomedical Engineering, Indian Institute of TechnologyMadras, Chennai, India; ^3^Laboratory for Human and Machine Haptics, Research Laboratory of Electronics, Massachusetts Institute of TechnologyCambridge, MA, USA; ^4^Department of Mechanical Engineering, Korea Advanced Institute of Science and TechnologyDaejeon, South Korea

**Keywords:** haptic exploration, movement stops, finger exploration, active touch perception, haptic perception process

## Abstract

**Introduction:** The explorative scanning movements of the hands have been compared to those of the eyes. The visual process is known to be composed of alternating phases of saccadic eye movements and fixation pauses. Descriptive results suggest that during the haptic exploration of objects short movement pauses occur as well. The goal of the present study was to detect these “explorative stops” (ES) during one-handed and two-handed haptic explorations of various objects and patterns, and to measure their duration. Additionally, the associations between the following variables were analyzed: (a) between mean exploration time and duration of ES, (b) between certain stimulus features and ES frequency, and (c) the duration of ES during the course of exploration.

**Methods:** Five different Experiments were used. The first two Experiments were classical recognition tasks of unknown haptic stimuli (A) and of common objects (B). In Experiment C space-position information of angle legs had to be perceived and reproduced. For Experiments D and E the PHANToM haptic device was used for the exploration of virtual (D) and real (E) sunken reliefs.

**Results:** In each Experiment we observed explorative stops of different average durations. For Experiment A: 329.50 ms, Experiment B: 67.47 ms, Experiment C: 189.92 ms, Experiment D: 186.17 ms and Experiment E: 140.02 ms. Significant correlations were observed between exploration time and the duration of the ES. Also, ES occurred more frequently, but not exclusively, at defined stimulus features like corners, curves and the endpoints of lines. However, explorative stops do not occur every time a stimulus feature is explored.

**Conclusions:** We assume that ES are a general aspect of human haptic exploration processes. We have tried to interpret the occurrence and duration of ES with respect to the Hypotheses-Rebuild-Model and the Limited Capacity Control System theory.

## Introduction

The sense of touch has already been described by Aristoteles (1986) and Weber (1846) as the most complex sensory system of men. Fundamental Experiments by von Skramlik (1937), Gibson (1962), and Revesz (1950) revealed that we have to distinguish between active touch (haptic perception) and passive touch (tactual perception). On account of its connection to motor processing, haptic perception is, among others, elementary for learning, body image, body schema, orientation in space, motor control, sexual activities, and perception of the blind (Schiff and Foulke, 1982; Heller and Schiff, 1991; Hatwell et al., 2003; Grunwald, 2008). Despite the huge importance of haptic perception for men, far more studies exist on the topic of passive, tactile stimulus perception. One reason for this may be methodological difficulties posed by the investigation of 10 finger tasks. Even though various studies concerning human haptic perception already exist, many aspects of information processing during haptic perception are still to be explained. Especially, the specifics of complex finger and body movements need to be investigated more thoroughly in healthy and unhealthy humans. Early on, the pioneers of haptic research (among others: Revesz, 1950 and Katz, 1925) have pointed out that it is crucial for the comprehension of human haptic perception, to understand how and with what kind of exploratory procedures surface and object characteristics are observed (e.g., with the fingers). Therefore, the precise analysis of exploratory procedures is essential to understanding the dynamics of movement and exploration during haptic perception. Accordingly, the analysis of these processes has the same significance as the precise analysis of ocular movements for the comprehension of visual perception. The exact knowledge of the interactions between visual scanning movements and cognitive stimulus processing has lead to substantial methodological as well as contentual progress in the field of visual research (Rayner, 1995; Kennedy, 1997; Walker, 1997; Inhoff et al., 2002; Vaughan, 2002; Findlay, 2005; Lansdown, 2005; Klein and Ettinger, 2008; Sui, 2008; Wade, 2010).

Concerning object exploration it is known that human haptic perception is accompanied by different touch movements, especially of the fingers. With the help of these active exploration movements, stimuli features are detected by different receptors (thermal receptors, vibration receptors, pain, and pressure receptors of the skin, muscles, tendons, soft tissue and joints). Furthermore, some studies have shown that exploration movements depend on task features as well as stimulus features (Lederman and Klatzky, 1987, 1993; Klatzky et al., 1993; Klatzky and Lederman, 1995). As early as in the middle of the last century, Ananev stated that touch and exploration movements of haptic perception include phases during which the fingers or hands hardly move or do not move at all (Ananev et al., 1959). He found that interruptions of movements occurred primarily on corners and edges. On a descriptive level it has, therefore, been known for quite a while that haptic explorative finger movements are interrupt by pauses. Lederman and Klatzky (1987) have described several “exploratory procedures” (EP) that were typically used by test subjects to explore object properties. To capture these global exploratory procedures, video footage was analyzed frame by frame. Their classification of EPs includes two static procedures: “static contact” and “unsupported holding.” The authors postulated that “static contact” is used to perceive object temperature and that “unsupported holding” is associated with the perception of object weight. The durations of these procedures were between 0.01 and 0.08 s for static contact and between 0.03 and 2.12 s for unsupported holding.

However, besides these global object EPs, little is known about the haptic perception of complex structures (e.g., raised-line pictures). The breaks and pauses that may occur during the exploration of complex haptic features have hardly been analyzed in healthy humans, yet.

In view of these facts, the consensus seems to be that haptic exploration is strongly linked with exploratory procedures. But it remains unclear to what purpose, why, when and for how long the explorative movements of the fingers stop. A theoretical and functional integration of explorative stops (ES) into the knowledge base of the haptic perception process is missing to date. That this problem has been addressed so little so far is all the more surprising as the direct comparison of explorative finger movements and eye movements is virtually obtrusive. More than half a century ago Russian psychologists formed first theoretical ideas that explorative hand and eye movements may be similar to each other (Zinchenko, 1957; Leontew, 1959; Zinchenko and Ruzskaia, 1962). A central aspect of this comparison concerns the scanning movements that are required for both the hands as well as the eyes to perceive. Alternations of saccadic movement and fixation periods that occur during the active visual process are well established. The oculomotor actions of vision are marked by a perpetual alteration between fixation pauses and saccadic eye movements. These fixation pauses are neither accidental occurrences nor an epiphenomenon of the oculomotor system. Results from cognition research and eye movement research have shown that the duration of the fixation pauses is associated with stimulus complexity (e.g., Krause, 1988; Kaller et al., 2009). The duration of the fixation pauses of the eyes increase with increasing complexity of the stimuli and, therefore, with increasing cognitive demands. Several theoretical concepts and empirical studies document a direct correlation of visual information processing and oculomotor acivity (Lüer et al., 1988; Liversedge and Findlay, 2000; Engbert and Kliegl, 2004; Martinez-Conde et al., 2004; Thomas and Lleras, 2007; van Gompel et al., 2007; Hutton, 2008).

Since we start with the premise that a psycho-physiological correspondence exists between the visual and the haptic system, it may follow that the exploration process of the human fingers may be composed of alterations of explorative movement and fixation periods as well. The present study was designed to capture possible ES of milliseconds during haptic exploration of various objects and surfaces. To make this possible, a measurement method with a higher temporal resolution than a video recording (frame-to-frame analyses) was necessary. Up to now, neither a digital measuring method which is able to measure the precise length of breaks during motion nor respective psycho-physiological studies have been reported. Therefore, we have developed a new measuring method to capture Experimental evidence for the existence of ES during the haptic exploration of objects and patterns.

**Figure 1:**
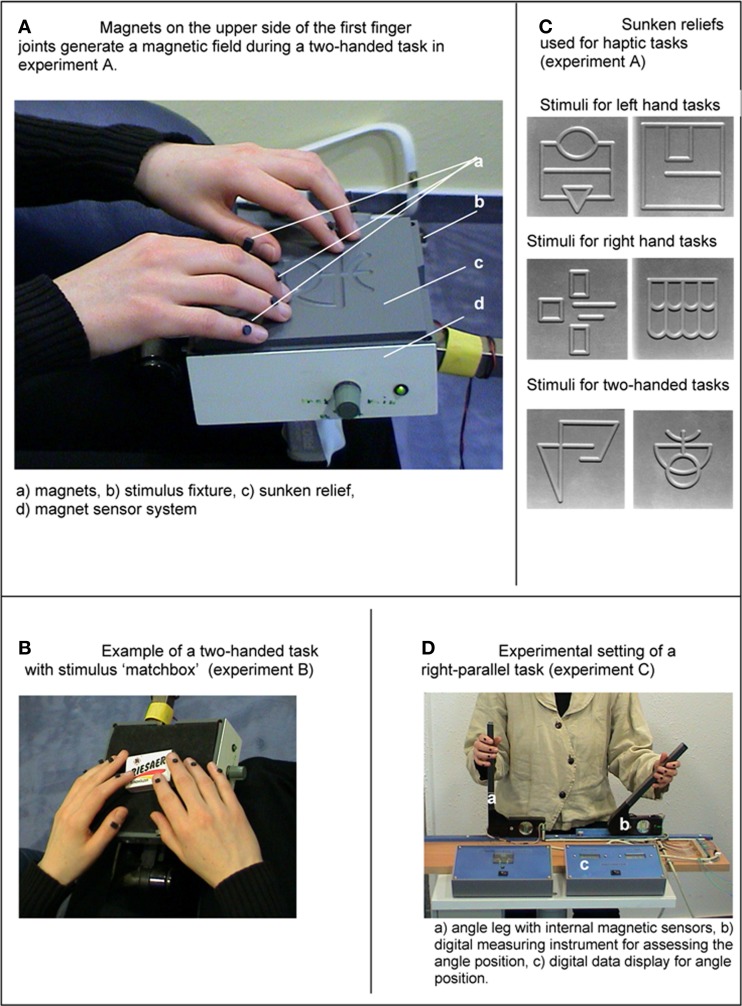
**(A)** Magnets on the dorsal side of the distal finger joints generate a magnetic field during a two-handed task in Experiment A. **(B)** Sunken reliefs used for haptic tasks (Experiment A). **(C)** Example of a two-handed task with stimulus “matchbox” (Experiment B). **(D)** Experimental setting of a right-parallel task (Experiment C).

The present study consisted of 5 Experiments (A–E). The first part of the study consists of Experiments A–C. The second part of the study (Experiments D and E) will be presented further below. Experiment A was used to test whether ES occur during the haptic exploration of sunken relief structures of unknown stimuli (Experiment A). In Experiment B the participants had to explore and recognize common objects. In Experiment C the spatial and angular position of angle legs had to be recognized and reproduced by the participants. The experimental settings and procedures are presented in Figures 1A–D. Further methodological descriptions are given in methods part one. We assumed that ES of milliseconds would occur during all three experiments (Hypothesis 1).

Furthermore, we expected to find, that ES would occur during uni- and bi-manual haptic exploration (Hypothesis 2). To test Hypothesis 2, Experiments A and B were conducted single-handedly as well as with both hands.

In correspondence with findings from eye movement research we assume, furthermore, that the mean duration of the ES is associated with the familiarity and complexity of the stimuli (e.g., Krause, 1988; Kaller et al., 2009). Therefore, the duration of the ES should increase with increasing complexity and novelty of the stimuli. To test this, the difficulty and complexity of the stimuli differed between Experiments A–C. The stimuli within each Experiment (A–C), however, were similar in their difficulty and complexity. Accordingly, we expect to find the shortest ES during the exploration of common objects. The longest ES should occur during the exploration of the unknown sunken relief stimuli. It is well established through visual research as well as haptic research, that reaction and recognition time are associated with the complexity of a task or stimulus (Krause, 1978, 1981; Lüer et al., 1988; Grunwald et al., 1999, 2001c). The more complex a stimulus is, the longer are the corresponding times. Therefore, the processing times pose as a direct measure of the internal cognitive information processing procedures. If this relationship exists for the ES as well, we expect to find a positive correlation of mean exploration time (ET) and mean duration of ES (Hypothesis 3). If, however, the ES are a random and reflexive occurrence that is unrelated to the stimulus properties, no association between ET and ES should be found.

To map the precise spatial locations of ES the PHANToM device was used in the second part of the study. As described above, the fixation pauses of the visual system do not occur accidentally, but are directly associated with information processing. Analogously, we assume that the ES of haptic perception depict cognitive information processing. Therefore, we assume that a spatial and temporal relationship exists between the occurrence and the duration of ES during haptic exploration and the stimulus properties. It is known from eye movement research that visual information processing occurs only during fixation pauses and not at all during saccadic eye movements. Correspondingly, we assume that the ES intervals represent phases of stimulus processing and sensory integration as well as aspects of motion control. Also, we assume that ES will not occur independently from the spatial structures of the stimulus features. We expect that ES will occur more often and with longer duration on stimulus areas which are high in information content (e.g., on corners, edges, and curves; Hypothesis 4). In the same line, ES should occur less often and with shorter duration on less complex stimulus areas like straight horizontal or vertical lines.

The perception of haptic patterns and objects is a serial process that requires gradual processing as well as cognitive integration of sensory motor information parts–similarly to visual stimulus processing. Therefore, we expect to find a temporal dynamic of (a) the frequency of ES occurrence and (b) the duration of ES during the course of haptic object and pattern recognition. Based on Richard Gregory's perception theory (Gregory, 1973) we believe that the haptic perception process consists of sequences of proposing and testing hypotheses until a final percept is generated. Therefore, we expect that the duration and frequency of ES will vary during the course of haptic exploration, especially on complex stimulus areas (e.g., corners) (Hypothesis 5). Specifically, we assume that the decoding of stimulus features at the beginning of haptic exploration will be accompanied by longer ES than the end of the exploration.

**Figure 2:**
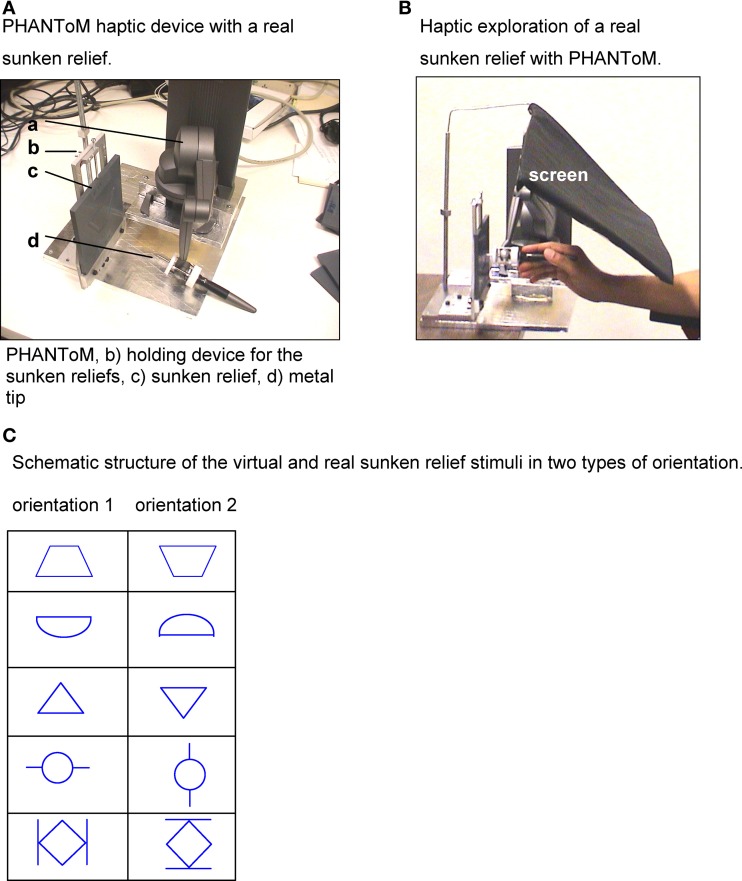
**(A)** PHANToM haptic device with a real sunken relief. **(B)** Haptic exploration of a real sunken relief with PHANToM. **(C)** Schematic structure of the virtual and real sunken relief stimuli in two types of orientation.

To test hypotheses 4 and 5 a technology was necessary that would facilitate a high resolution analysis of the spatial stimulus features as well as of the temporal course of haptic exploration. Therefore, the PHANToM haptic device was used in Experiments D and E. The participants had to explore virtual and real sunken reliefs with the tip of the PHANToM device (Figures 2A–C; see methods part 2). Both virtual and real stimuli were explored with the help of the PHANToM device to assess whether differences exist in the exploratory procedures.

## Materials and methods

### Part 1

#### Measurement methods for experiments A–C

During the haptic tasks of Experiments A–C finger and hand movements were measured by a digital apparatus, which is designed for measurements of smallest changes within a magnetic field. The apparatus consisted of three linked, highly sensitive magnetic sensors (sensor type: KM 110BH/2310, Philips Semiconductors U.S.). The magnetic sensors were located within the stimulus fixture in Experiments A and B (Figure 1), whereas in Experiment C, the magnetic sensors were located within the moveable angel legs. The sensitive measuring range of the sensors amounted to 9 cm. Small magnets (3 mm in diameter), which were glued to the dorsal side of the distal interphalangeal joints of the test persons, generated a measurable magnetic field. During finger and hand movements the magnetic field changed, and the electric output of the magnetic sensors varied between 0 and 300 μ V. Whereas, during an absolute motionless state the electric output varied only between 0 and 1 μ V. Therefore, this measurement method had a very high temporal resolution. The output signals were recorded with a sampling rate of 166.66 Hz and were saved digitally. To record the measurements a digital EEG device (Walther Graphtek, Munich, Germany) was used. The measurement of ET began when the hands first touched the stimuli (Experiment A and B) or when they first touched the angel legs (Experiment C). The output signals of the magnetic sensors were recorded separately for each angle leg (Channel 1, 2). The analysis of the output signals was carried out with the software BRAIN VISION (Munich, Germany). Signals within a range of 0–1 μV were marked as ES. Output signals >1.0 μV were marked as motions.

#### Experiment A

The test persons had to explore (with their fingers) the structure of different sunken reliefs while their eyes were closed. The structure of the reliefs consisted of milled traces with a depth of 3 mm and a width of 7 mm (Figure 1B). Optimal positioning of the stimuli in relation to the fingers was ensured by an adjustable holder. During haptic exploration the participants' forearms rested on a wide base in order to allow free movements of the fingers only. No arm and shoulder movements were made during haptic exploration. The ET per stimulus was not limited. After haptic exploration the participants were asked to open their eyes and to draw the perceived structure on a piece of paper. The test persons were prevented from gathering any visual information from the stimuli. They received no feedback on the quality of their reproduction or on the stimulus structure itself. Before the Experiment proper began, the participants were allowed to look at and explore a sample stimulus (that was not included in the following Experiment) to become familiar with the haptic material. They practiced the exploration task for 1 min.

Three task types were distinguished: left hand tasks, right hand tasks and two-handed tasks. Each participant had to complete all tree tasks. To complete each task the participants had to explore and draw two sunken reliefs. In other words, they explored two sunken reliefs with the left hand, two with the right hand and two with both hands, consecutively. For the three task types different haptic stimuli were used to prevent memory effects (Richardson and Richardson, 1996). Based on the study by Ballesteros et al. (1997) we used one symmetrical sunken relief and one asymmetrical sunken relief for each task. The same haptic stimuli of sunken reliefs have been used before in psycho-physiological and clinical Experiments to investigate brain electrical changes, e.g., in patients with Anorexia nervosa (Grunwald et al., 2001a,b), Alzheimer's Disease (Grunwald et al., 2002a) and healthy participants (Grunwald et al., 1999, 2001c).

#### Experiment B

In Experiment B the participants had to explore and to recognize 15 common objects: 5 objects with the left hand (left hand tasks), 5 objects with the right hand (right hand tasks), and 5 objects with both hands (two-handed tasks). The following stimuli were used: corkscrew, pen, note-book, walnut, screwdriver, battery, toothbrush, glasses, candle, eggcup, crown cap, matchbox, cigarette lighter, woodscrew, blister pack (Figure 1C). The order of the stimuli as well as the order of the tasks varied between the test persons. (Some test persons started with right hand tasks, some with left hand tasks and so on.) No time limit was set for haptic exploration. While exploring, the participants' eyes were closed. Additionally, a shield prevented participants from seeing the stimuli. An acoustic signal indicated that participants should start with the haptic exploration. They were allowed to move and explore freely without restrictions as long as they did not lift the stimuli from the holder. In that moment when the test persons recognized the common object they were to take the hand (or hands) away from the stimulus and name the object. Figure 1C shows an example of a two-handed task with the stimulus “matchbox.” Before the Experiment began the participants performed a training trial with a pair of scissors (training stimulus) to get familiar with the course of the Experiment.

#### Experiment C

In Experiment C we used the experimental setting of the Angle Paradigm as outlined in Figure 1D. The experimental setting of the Angle Paradigm has already been used in several studies (Grunwald et al., 2002b; Grunwald and Weiss, 2005). The design consisted of two angle legs, of which one angle leg had to be adjusted by the participant. We distinguished between two task types—right parallel task and left parallel task. For the right side task the *left* angle was locked at a certain angle position and the participant was asked to bring the right angle leg in a parallel position to the locked left angle leg. In contrast, to solve the left side tasks the left angle had to be adjusted to the locked right angle leg. Each task consisted of five different angular positions. No time limit was given and no visual feedback was provided. The starting position of the angle legs that had to be adjusted by the test subjects was 90°. All participants performed two training trials to become familiar with the assignment. After the training trials the participants were given visual as well as verbal feedback about their results in form of degrees of deviation.

Afterwards, the participants were blindfolded while their hands rested on touch sensitive switches. These switches indicated when the participant began moving toward the angle legs. Then, the experimenter prepared the first task. Figure 6 shows the left angle leg (as seen by the test person) which was adjusted to a defined angle (nominal value) by the experimenter. The right angle leg had a starting position of 90°. Next, the participant was asked to bring the right angle leg in a parallel position to the left (target) angle leg. Then, the experimenter noted the adjusted angle (actual value) and prepared the next task. Nominal values for the right side tasks were: 135, 158, 125, 165, and 145°, the nominal values for the left side tasks were: 45, 22, 65, 15, and 35°. All participants had to solve the tasks of one task type in the same order, but the order of the task types varied.

During the exploration, the left hand was only allowed to touch the left angle leg, and the right hand was only allowed to touch the right angle leg. No cross-over or both-handed exploration or touching of the opposite angle leg and the tabletop was allowed. Both hands should leave the table and explore the angle positions simultaneously. The measurement of ET started with the first contact of the hands and the angle legs. The target angle leg was not moveable by the test person. The exploration of the angle legs was performed through various up-and-down movements of one or more fingers along the angle legs. The participants were asked to return their hands to the starting position on the table as soon as they finished a task.

The angle position was assessed by a digital measuring instrument with an accuracy of one hundredth of a degree, provided by the company NESTLE (Dornstetten, Germany). Additionally, the deviations of the angles were shown on a separate display.

Two hollow metal bars (5 mm × 10 mm × 240 mm) served as angle legs. The distance from the table to the end of the angle legs was 28.7 cm in the position of 90°. The distance between the angle pivots was 28 cm. After the angles were adjusted the angle data was recorded manually by the experimenter. ETs (the time needed for the adjustment of the angle leg), duration and number of ES were assessed.

### Part 2

#### Measurement methods for experiment D (virtual)

The participants had to explore the structure of virtual sunken reliefs with the PHANToM device. Their eyes were closed. During the exploration the participants held the PHANToM device in their right hand, in standard position. They were able to move their hand and forearm freely. The participants could choose their individual starting position for each stimulus. The PHANToM device generates the virtual stimulus by giving force-feedback signals while the participant moves the device through the air. No time limit was set for the haptic exploration. No visual feedback was given on the stimulus structure at any point of the Experiment.

The stimuli had a virtual size of 13 × 13 cm. Their structure resembled milled traces of 3 mm depth and 7 mm width. To prevent the tip of the PHANToM device from slipping off the sidewalls, they were programmed as 6 cm high walls. The virtual stimuli were constructed with the program package Autodesk 3D MAX. The actual sunken relief stimuli that were used in Experiment E served as a data base.

#### Measurement methods for experiment E (real)

The participants had to recognize the structure of real sunken reliefs stimuli (one example see Figure 2A) with explorative movements of a metal tip. The metal tip was fixed to the upper end of the PHANToM device (see Figure 2A). Their eyes were closed during the procedure. No visual feedback was given at any time of the Experiment. The stimuli consisted of hard plastic boards of 13 × 13 × 0.5 cm with a relief structure of milled traces with a depth of 3 mm and a width of 7 mm. All test persons held the PHANToM device in their right hand. Neither a starting position nor a time limit was given for the exploration. For the exploration, the sunken relief stimuli were fixed in a solid holding device. To prevent participants from gathering any visual information of the stimulus, not even by chance, a screen was strategically placed.

#### Experiments D and E

A *Phantom Desktop* (Sensable Technologies, USA) was used, with six-degrees-of-freedom positional sensing, Nominal position resolution: >1100 dpi, ~0.023 mm, Force feedback (3° of Freedom; x, y, z), Stiffness: x-axis > 10.8 lb/in (1.86 N/mm); y-axis > 13.6 lb/in (2.35 N/mm); z-axis > 8.6 lb/in (1.48 N/mm). Therefore, a precise mapping of the spatial positions of the ES was possible with this device.

The experiments encompassed five sunken relief stimuli that were presented twice (virtual stimuli in Experiment D and real stimuli in Experiment E, as described above). The stimuli were presented in standard orientation (0°) (orientation 1) and then the same five stimuli were presented again, but turned by 180° (orientation 2). The order of the presented stimuli varied for each participant. For each stimulus, corrected x-, y-, and z- coordinates of the PHANToM device (that means the position of the metal tip during the exploration) were recorded with a sampling frequency of 1 KHz and stored digitally. During the exploration the participants could move their hand and forearm freely (they did not lie on a base). After the exploration process, the participants were asked to open their eyes and to draw the perceived sunken relief pattern on a piece of paper. After they finished drawing, the participants closed their eyes again and the next stimulus was presented.

Before the Experiment began, a test stimulus (real/ virtual) was presented and the experimental task, the PHANToM device and its operations were explained. During a 10 min training presentation the participants were free to open and/or close their eyes to get familiar with the stimulus structure and the task.

#### Reference study to experiments D and E

Due to the peculiarities of performing haptic tasks with the PHANToM device, we assessed which characteristic movements occur during haptic perception of a horizontal line under virtual and real conditions. These reference studies were performed prior to the actual Experiments. We used these tasks as references because they require less perceptual cognitive processes with the most important being motor control. To perform the reference task the participants (*n* = 10) touched a single horizontal line with the PHANToM device for 5 min. Their eyes were closed. The subjects were informed that their task would be to repeatedly follow the horizontal line and that the resulting measures would be used as reference values. Two conditions were used: First, the participants were presented a real sunken relief line; secondly, a virtual sunken relief line was presented. The scanning velocity during this test differed from subject to subject. All participants generated motion stops with a mean length of *M* = 89 ms (*SD* = 40 ms). These stops occurred only at the end points of the line (left or right side, under virtual and real condition). These kinds of stops were termed “mechanical stops.” Since no theoretical basis for the discrimination between mechanical and ES based on duration exists, the somewhat artificial value of 89 ms was used to discriminate between ES and mechanical stops during the PHANToM Experiments. The cut-off was generated merely to account for the technical limitations of the exploration movements due to the PHANToM device. Therefore, for Experiments D and E only those motion stops that lasted longer than 89 ms were marked as ES and used for data analysis.

During manual haptic exploration pure motor stops may possibly occur as well. However, again, there is no theoretical basis for discriminating between motor and ES at the current stage of research. The present study is the first to explore the mere existence of stops during the haptic exploration process. The determination of possible subgroups of stops needs to be left to future studies. Since no technical movement limitations exist during manual haptic exploration and since there is as much reason to assume that ES that are shorter than 89 ms exist just the same, no such cut-off was used for Experiments A–C.

### Participants

#### Experiments A–C

The same eight healthy volunteers (4 men, 4 women) took part in all three Experiments (A–C). All participants were right-handed according to a test of handedness by Salmaso and Longoni (1985). After all test persons had been fully informed about the aim and content of the investigation, written consent was obtained. The participants received 10 € compensation for each session. The study was approved by the local Ethics Committee of the University of Leipzig (Germany).

#### Experiments D and E

Ten test persons (5 women, 5 men) took part in the investigation. The participants were students and assistants of the Research Laboratory of Electronics (RLE, MIT, Boston USA). All participants were right-handed according to a test of handedness by Salmaso and Longoni (1985). Between the execution of Experiments D and E a 4 weeks waiting-period was met by all participants. Each participant received a monetary compensation of 10$ for each session. All participants were informed about the aims of the study and gave their written consent. The study was approved by the local Ethics Committee at the Massachusetts Institute of Technology (Boston, USA).

### Statistics

For all analyses statistics software SPSS 20.0.0 was used. For statistical comparisons between tasks (Experiments A–E) ANOVA were calculated. For the statistical comparisons between task types (orientation 1, 2) paired *t*-tests (critical alpha 0.05) were used. The standard Pearson correlation coefficient (one tailed) was used to assess the correlations between ET and length of ES.

## Results

### Part 1

The analysis of the data revealed that during haptic exploration of sunken reliefs (Experiment A) several ES of on average 300 ms occurred. ES were observed during one-handed as well as two-handed exploration (Table 1). During the exploration of common objects (Experiment B) stops with an average length of 70 ms occurred (Table 1). Again, stops were observed during one-handed as well as two-handed tasks. Explorative movements during the “angle paradigm” (Experiment C) were also interrupted by ES, which had an average length of 190 ms (Table 2). Thus, for haptic exploration, it could be demonstrated that the fingers persisted in a static position on the stimulus during phases of ES during all three task types (Hypothesis 1) as well as during one- and two-handed exploration (Hypothesis 2). The number and duration of ES did not differ between one- and two-handed tasks.

**Table 1:** **Descriptive data and ANOVA results of Experiment A (sunken reliefs) and Experiment B (common object exploration)**.

	**Experiment A**					**Experiment B**				
	**Left hand tasks**	**Right hand tasks**	**Two-handed tasks**	***F*_(2, 21)_**	***p***	**Left hand tasks**	**Right hand tasks**	**Two-handed tasks**	***F*_(2, 21)_**	***p***
ES *M* (*SD*) min-max	0.36 (0.23) 0.08–0.98	0.32 (0.12) 0.05–0.52	0.30 (0.15) 0.09–0.62	0.47	0.629	0.08 (0.07) 0.02–0.22	0.04 (0.03) 0.01–0.12	0.06 (0.06) 0.02–0.18	0.75	0.481
*N* Stops *M* (*SD*)	32.50 (22.53)	43.31 (31.86)	28.05 (21.20)	1.05	0.361	6.75 (6.08)	5.25 (5.49)	4.62 (3.15)	0.37	0.695
ET *M* (*SD*)	138.14 (47.50)	216.67 (118.70)	268.12 (162.85)	2.73	0.008	5.06 (6.85)	4.58 (1.47)	4.75 (6.85)	0.10	0.899

Mean Time of explorative stops (ES) in seconds, mean number of explorative stops (N Stops), mean time of haptic exploration (ET) in seconds.

**Table 2:** **Descriptive data for Experiment C (Angle Paradigm)**.

	**Experiment C**			
	**Right parallel tasks**	**Left parallel tasks**	***F*_(1, 14)_**	***p***
ES *M* (*SD*) min–max	0.12 (0.07) 0.05–0.29	0.25 (0.12) 0.17–0.54	6.403	0.024
*N* Stops *M* (*SD*)	10.12 (11.98)	11.12 (13.06)	0.025	0.876
ET *M* (*SD*)	25.59 (14.79)	23.49 (13.72)	0.087	0.773

Mean Time of explorative stops (ES) in seconds, Mean number of explorative stops (N Stops), mean time of haptic exploration (ET) in seconds and ANOVA results.

**Figure 3:**
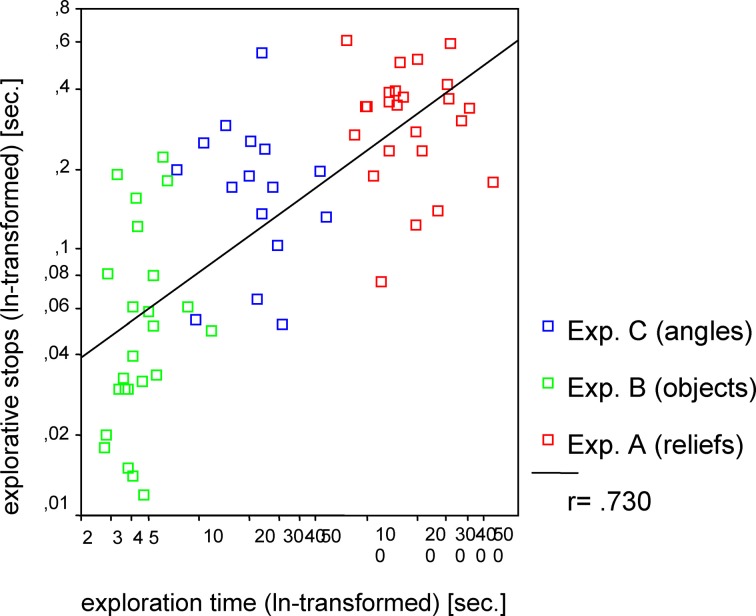
**Correlation between exploration time and explorative stops for Experiments A–C**. Pearson correlation (one tailed) was used.

Hypothesis 3 was also confirmed. Remarkably, the mean length of the ES differed significantly between the three experimental conditions [*F*_(2, 61)_ = 34.05, *p* < 0.001]. The shortest average length of ES was measured during the exploration of common objects, whereas the longest average length of stops occurred during the exploration of sunken reliefs. To calculate the correlative relationships between length of ES and ET the data was transformed (logarithmized). The standard Pearson correlation revealed a significant correlation (*r* = 0.730, *p* < 0.001; see Figure 3) of ES and ET. Accordingly, the mean length of ES increased with the mean duration of the ET (Hypothesis 3).

### Part 2

**Figure 4:**
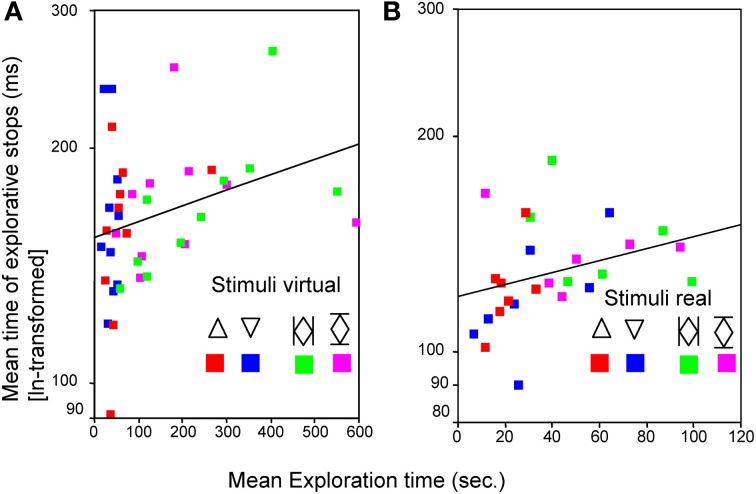
**Relationship between mean time of explorative stops (ms) and the mean exploration time (s) for a simple** 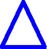 **and a complex** 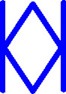 **stimulus in both orientations (1 and 2) for virtual (A) and for real (B) stimuli**. Pearson correlation (one tailed) was used.

During the two PHANToM experiments (Experiment D with virtual sunken reliefs and Experiment E with real sunken reliefs) ES were observed as well. ES with a mean length of *M* = 186.17 ms were measured in Experiment D (virtual condition) and of *M* = 140.02 ms in Experiment E (real condition). No differences in mean length of ES, number of ES, and mean ET (see statistical results in Table 3A) were found between the two orientation condition (stimulus orientation 1, 2). But, as expected, significant differences existed between virtual and real stimuli. ETs as well as the length of the ES were much shorter in Experiment E (real sunken relief stimuli) than in Experiment D (virtual stimuli). Furthermore, significantly fewer ES occurred in Experiment E than in Experiment D (see Table 3B. Additionally, a linear correlation between ET and mean length of ES was found for Experiments D and E. This result is in line with the correlation found across Experiments A–C. The correlation coefficient was *r* = 0.289, *p* = 0.035 for virtual stimuli (Experiment D), and *r* = 0.331, *p* = 0.043 (Pearson, one–tailed) for real stimuli (Experiment E) (see Figure 4). That means that with increasing ET also the mean length of ES increased in both experiments. Therefore, Hypothesis 3 was confirmed for the PHANToM experiments D and E as well.

**Table 3a:** **Description of experimental data from Experiment D (virtual) and Experiment E (real)**.

	**Virtual**				**Real**			
	**Stimuli orientation 1**	**Stimuli orientation 2**	***t***	p^*^	**Stimuli orientation 1**	**Stimuli orientation 2**	***t***	p^*^
Mean time explorative stops [ms] *M* (*SD*)	182.26 (28.83)	190.08 (39.45)	0.774	0.459	141.55 (22.37)	138.50 (32.29)	0.621	0.537
Mean exploration time [s] *M* (*SD*)	114.79 (112.08)	90.13 (95.14)	2.16	0.036	37.03 (20.21)	35.19 (24.67)	0.431	0.669
Mean number of stops *M* (*SD*)	29.98 (34.56)	22.80 (29.39)	1.84	0.071	10.88 (7.01)	9.66 (7.78)	0.931	0.356

Statistical comparison between orientation 1 and orientation 2.

* Significance level paired t-test.

**Table 3b:** **Description of experimental data and statistical comparison between virtual stimuli (Experiment D) and real stimuli (Experiment E)**.

	**Experiment D**	**Experiment E**	***t***	p^*^
	**Virtual**	**Real**		
Mean time explorative stops[ms] *M* (*SD*)	186.17 (33.86)	140.02 (27.68)	2.636	0.027
Mean exploration time [s] *M* (*SD*)	102.46 (104.17)	36.11 (22.46)	3.851	0.004
Mean number of stops *M* (*SD*)	26.39 (32.12)	10.27 (7.40)	2.432	0.038

* Significance level paired t-test.

**Figure 5:**
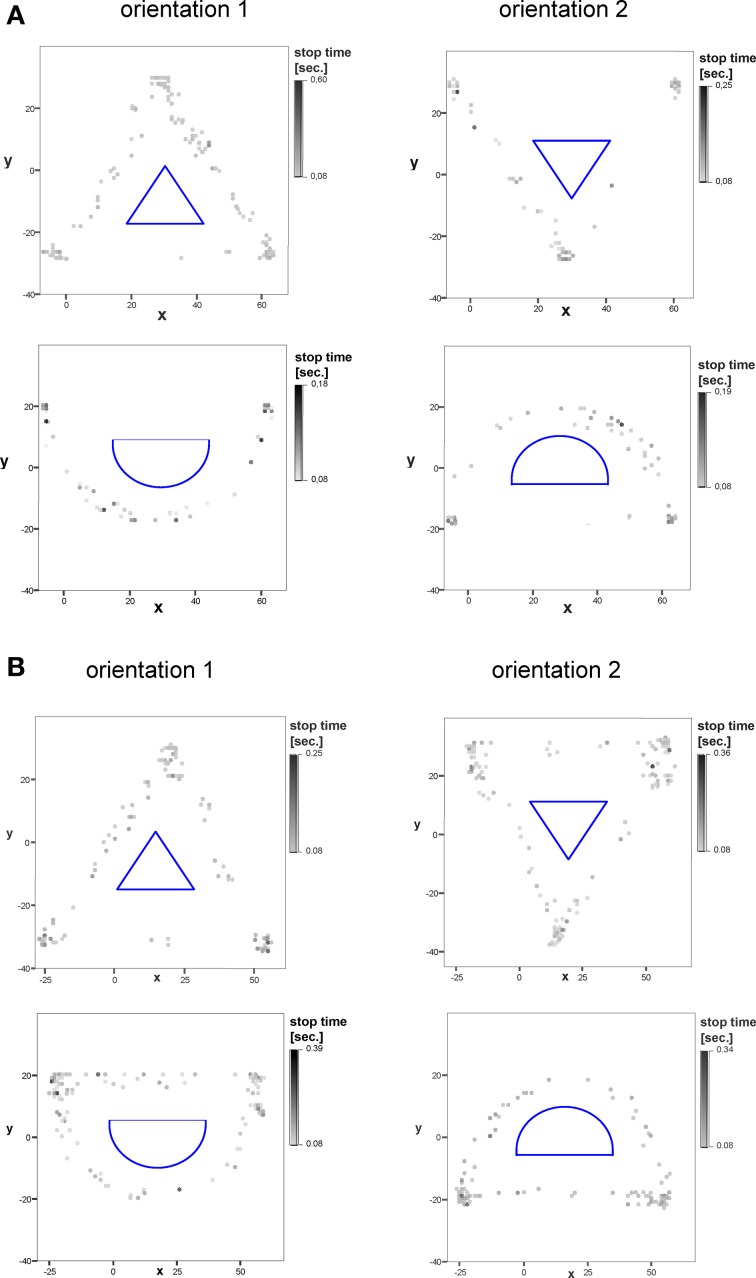
**Example of the allocation and xy-position of explorative stops of two stimuli in the virtual (A), and real (B) test condition in orientation 1 and orientation 2 (10 subjects)**. The duration of explorative stops is marked in different grayscales. The stimulus structure is displayed in the middle (blue).

The analysis of how ES are distributed spatially during the haptic exploration of virtual and real sunken reliefs (with the PHANToM device) revealed that ES vary in frequency at different stimulus features (Hypothesis 4). ES occur more frequently at corners, endpoints of lines and on curves (see Table 4), whereas fewer ES were observed on vertical and horizontal lines. For an example of the spatial distribution of ES in Experiment D and E please see Figure 5. The mean ES length did not differ between the different stimulus features (Table 4), neither for virtual stimuli [*F*_(6, 71)_ = 1.175, *p* = 0.330] nor for real stimuli [*F*_(6, 78)_ = 0.393, *p* = 0.882]. The number of ES per stimulus feature did differ significantly, however, in both experiments [*F*_virtual(6, 71)_ = 6.228, *p* < 0.001; *F*_real(6, 78)_ = 9.094, *p* < 0.001; Table 4].

**Table 4:** **Number (No) of explorative Stops (ES), and mean time of ES in relation to stimulus features for Experiment D (virtual sunken relief) and experiment E (real sunken relief)**.

	**Virtual**				**Real**			
			**ES in ms**				**ES in ms**	
	**No**	**%**	***M***	***SD***	**No**	**%**	***M***	***SD***
Corners	205	35.2	130.77	13.16	474	46.4	147.95	30.13
Endpoints of lines	165	28.3	148.81	65.40	177	17.3	139.05	21.24
Vertical lines	4	0.7	119.50	0.0	29	2.8	147.12	20.66
Horizontal lines	3	0.5	94.00	0.0	25	2.4	132.68	36.21
Circles	87	14.9	126.95	20.44	54	5.3	141.52	56.04
Cross points	55	9.4	129.59	22.05	192	18.8	135.19	20.88
Sloping lines	64	11.0	137.74	32.72	70	6.9	132.57	45.12

**Figure 6:**
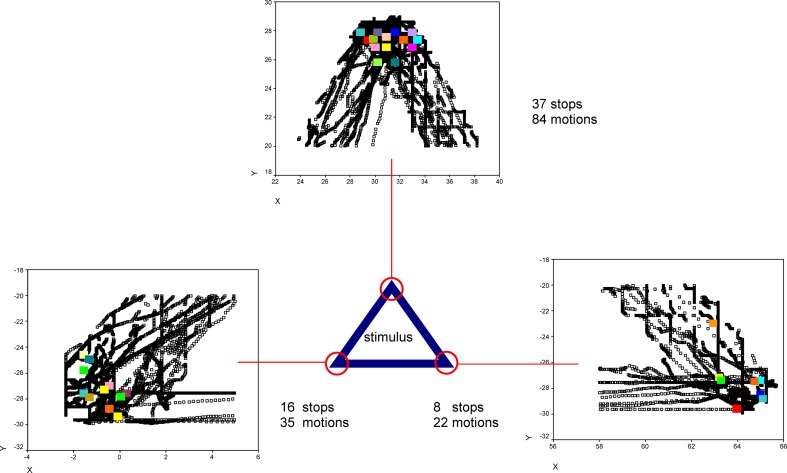
**Exemplary XY-positions of one participant's explorative stops (color points) and motions (black points) at the corners of one virtual stimulus (triangle) are shown**. Under/ beside the diagrams the number of explorative stops and the number of motions is indicated.

An additional, explorative analysis revealed that the number of ES differed from the number of motions. That means, that ES did not occur during every haptic motion at every stimulus feature, as exemplary outlined in Figure 6.

**Figure 7:**
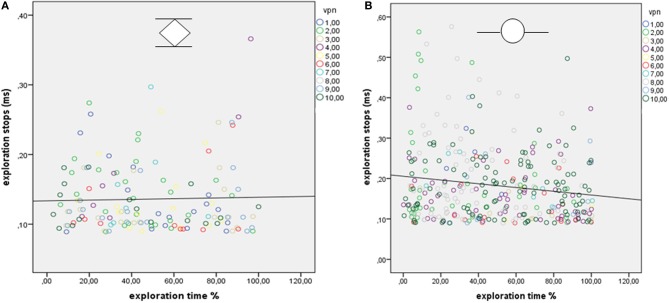
**(A,B)** Regression plots show the relationship between relative exploration time and duration of explorative stops (ES) for one stimulus from experiment E [real sunken reliefs; 10 participants (VPN)] and **(B)** for one stimulus from Experiment D (virtual sunken reliefs, 10 participants).

To investigate Hypothesis 5 (whether the duration of ES varied during the exploration process) the *relative* ET and the duration of ES were correlated per stimulus. We expected to find a systematic decrease of stop duration toward the end of the ET. The Pearson correlations (one-tailed) revealed divergent and non-significant results. Both positive and negative correlations occurred, that did not reach the critical alpha value (α = 0.0025, Bonferroni correction for multiple comparisons). The results are presented in Table 5. Exemplary regression plots for one real and one virtual stimulus are displayed in Figure 7.

**Table 5:** **Pearson correlation (one-tailed) between relative exploration time and length of ES (explorative stops in ms) per stimulus for Experiment D (virtual sunken relief) and Experiment E (real sunken relief)**.

**Stimuli**	**Real stimuli**			**Virtual stimuli**		
	***r***	***p***	**Number of ES**	***r***	***p***	**Number of ES**
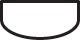	0.138	0.069	117	−0.061	0.268	106
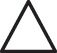	0.088	0.259	56	−0.156	0.027	153
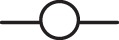	0.039	0.309	165	−0.151	0.003	333
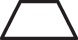	−0.158	0.030	143	−0.093	0.067	258
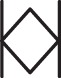	−0.044	0.281	175	0.019	0.319	641
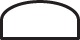	0.143	0.073	104	0.045	0.308	128
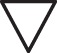	−0.193	0.034	90	0.069	0.246	101
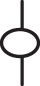	0.083	0.205	101	0.107	0.057	218
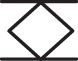	0.109	0.006	528	−0.060	0.200	200
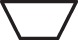	−0.027	0.363	176	−0.070	0.070	445

## Discussion

All five experiments demonstrated that the haptic exploration movements include ES of milliseconds. Thus, basically, the haptic exploration process (with closed eyes) may be regarded as an alternating cycle of explorative motions (EM) and ES. During haptic exploration of unknown sunken relief stimuli (Experiment A) ES with a mean duration of 329.50 ms occurred, whereas during haptic exploration of common objects (Experiment B) ES lasted only 67.47 ms, on average. The average duration of ES during the processing of space-position information (angle leg adjustments, Experiment C) was 189.92 ms. Mean ES of 186.17 ms were observed during the exploration of virtual sunken reliefs (Experiment D) with the PHANToM haptic device. ES of 140.02 ms, on average, were found during the exploration of real sunken reliefs (Experiment E) which were touched with the PHANToM device. ES were observed during one-handed as well as two-handed tasks. The results confirm the hypothesis that human haptic perception is generally accompanied by movement pauses of the exploring fingers and hands in healthy humans.

A strong correlation was revealed between mean duration of ES and mean ET per stimulus (see Figure 3). Short ETs coincided with shorter ES. In contrast to this, ES lasted significantly longer during longer ETs. Therefore, the duration of ES is not independent from ET. The same correlation was also found in Experiment D and E. Ergo, the correlation of mean ET and length of ES was found for both virtual and real stimuli, during both manual and PHANToM exploration. The stimuli of the different experiments differed in complexity. As introduced above, ET poses as an indicator of information processing and cognitive demands. According to studies from Rösler et al. (1993) and Grunwald et al. (1999) ET varies depending on the perceptive-cognitive processing effort during haptic exploration. We found that longer ETs and increasing stimulus complexity coincided with a longer average duration of the ES. The shortest ES were measured during the haptic exploration of common familiar objects. Thus, the strong correlation between mean ET and duration of ES may be understood as the perceptive-cognitive processing effort during information integration. Similar results, showing that stimulus complexity and the duration of fixation pauses are correlated, have been presented for the visual modality (Krause, 1988; Kaller et al., 2009).

To answer the question where and at which stimulus features ES occur, we used an experimental design and apparatus (Experiment D and E) which allowed us to precisely register the Cartesian coordinates (x, y, z) of the haptic exploration process. The PHANToM haptic device makes haptic perception in virtual space possible (Salisbury and Srinivasan, 1997). In Experiment D the participants had to recognize five different virtual sunken reliefs with the PHANToM device while their eyes were closed. To compare virtual and real stimuli, the virtual sunken reliefs of Experiment D were presented as real sunken reliefs in Experiment E. The test persons had to explore these real sunken reliefs with a special one-point-stick that was mounted to the end of the PHANToM holding device (see methods Part 2).

In both cases the touch perception with the PHANToM device presents a profound reduction of the natural dimensions of haptic perception. Natural haptic perception should be considered as far more complex, as it is not restricted to the information from one single point as the haptic perception with PHANToM is. Despite these limitations, haptic perception with the PHANToM device is roughly comparable to the haptic perception of a single finger or with a handheld pen.

The spatial distribution of ES in Experiment D and E showed that ES occur more frequently at certain stimulus features (i.e., corners) in contrast to other features (i.e., lines). However, ES did occur on all stimulus positions; not only on cross and end points. Also, the analysis showed that salient stimulus features are more frequently explored than ES occur. That means that ES do not occur every time the finger moves along the stimulus feature. Thus, the number of haptic motions that may be observed at a certain stimulus feature may be higher than the number of ES that occur at the same feature. This characteristic indicates that not stimulus features themselves are responsible for ES, but that the occurrence of ES is more likely to be related to the perception process—possibly even to information processing.

Hypothesis 5 was based on the assumption that the participant generates a hypothesis about the whole stimulus right from the beginning of the exploration process. Therefore, the ES were expected to be longer at the beginning of the exploration than at the end, because more new information has to be processed at the beginning than at the end of the exploration. The assumption implies that the amount of information that has to be processed corresponds with the duration of the stops. However, the supposition ignored the differential exploration properties of the PHANToM device as opposed to natural 10 finger exploration (Experiments A–C). The exploration with the PHANToM device consists of only one contact point with the stimulus and, therefore, only one-point information. Consequently, it is not possible to generate a hypothesis about the whole stimulus at the beginning of the exploration during Experiments D and E.

**Figure 8:**
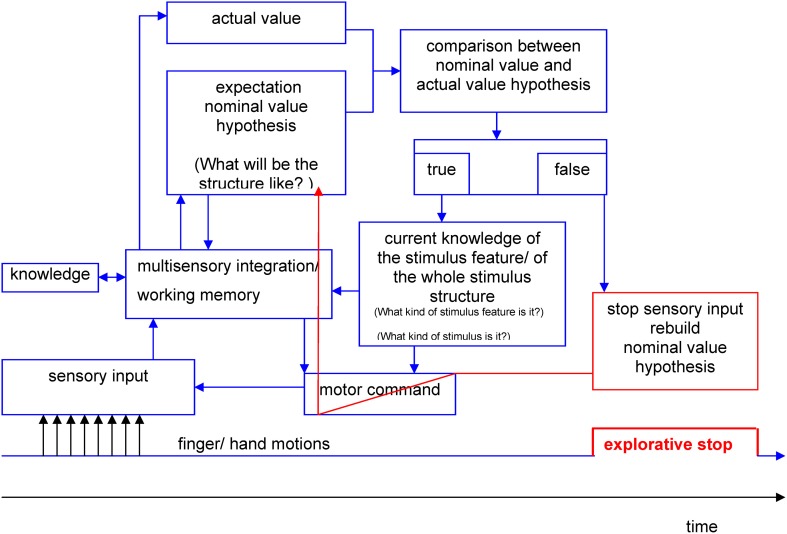
**Hypothesis-rebuild-model for explorative stops during human haptic perception (details see text)**.

Therefore, we are not surprised that the assumption of a systematically negative correlation between stop duration and temporal position during the exploration process had to be dismissed. The temporal allocation of ES and stop duration showed positive as well as negative correlative associations at low significance levels for different stimuli. Additionally, a temporally stable distribution of ES was observed across the exploration process. These findings (the occurrence as well as the dynamics of ES) may still be in line with the “hypotheses-rebuild-model” (see Figure 8), however. In this model, perception is understood as an active constructional process and not as a passive observation of environmental stimuli. Analogous to Richard Gregory's perception theory (Gregory, 1973) the haptic perception process may consist of sequences of proposing a hypothesis and testing the hypothesis. Hypotheses about the expected structure of stimulus features (nominal value) are serially compared with incoming information about actual stimulus features (actual value) by bottom-up as well as top-down processing.

During the first phases of the perception process the hypotheses are pre-attentive. If there are no differences between the expected value and the actual value the result of the comparison will be stored. This process lasts until a difference is stated between actual and nominal value on a conscious level. If an unexpected stimulus feature occurs (e.g., corner instead of line) the nominal value hypothesis has to be corrected. For the proposition of new nominal value hypotheses only limited processing resources of working memory are available. The necessary resources are regulated by the limited capacity control system (LCCS) (Gopher and Donchin, 1985). A possible consequence of nearly exhausted processing resources may be that the further income of sensory information is put on halt. Explorative movements may come to a standstill during the reorganization of working memory resources, which may be measurable as ES. The results of the present study showed, that a temporally stable distribution of ES across the exploration process occurred. This may be due to continuously incoming information that needs to be processed by working memory. Likewise, a continuous generation of hypotheses about the expected actual values is necessary during the one-point exploration with the PHANToM device.

The hypothesis-rebuilt-model may also serve as an explanation why ES are shorter during the exploration of common objects (see Experiment B) than during the exploration of unknown objects. The degrees of freedom for hypotheses about common objects may be limited by existing knowledge. And thus, hypotheses are generated faster and less information has to be stored in working memory.

Furthermore, the model may even be fit to explain why ES do not occur *per se* at complex stimulus features (i.e., cross points). In terms of Gregory's perception theory, sensory income would only be interrupted at those features for which the internal hypotheses are not validated yet. In terms of the hypotheses-theory the occurrence of ES would be a function of a perceptive-cognitive test process. However, the validity of this model cannot be clarified by the results of the present study. Future studies need to examine whether the frequency and/ or duration of ES systematically changes after unforeseeable changes of the stimulus structure (e.g., virtual stimuli) during the exploration process. In that case, the duration and number of ES should increase with each structural change of the stimulus because the participants would have to constantly adjust their hypotheses.

For the time being, individual variations that may be due to different explorative strategies and differences in processing time cannot be explained conclusively by the present results. Furthermore, the methodological limitations of the PHANToM device call for the analysis of temporal and spatial characteristics of ES during 10 finger tasks in future studies. Nevertheless, during the present study ES were observed during the haptic exploration of a wide variety of stimuli. Therefore, it feels save to assume that ES are a stable aspect of human haptic perception.

Future studies may evaluate the possible relevance of ES for diagnostic purposes. Possibly, differences in the distribution, frequency and duration of ES may be found for people with different kinds of psychiatric disorders or cognitive strategies.

With the help of electrophysiological parameters (EEG, MEG or fMRI) further studies may reveal corresponding cortical processes of touch motions and of ES during human haptic perception. Spectral EEG analyses of the theta-band may elucidate whether ES are associated with working memory consolidation. If that is the case, a significant increase of theta would be expected. If ES are accompanied by hypotheses-rebuild-processes, on the other hand, increases of beta and gamma frequencies may be more likely. Besides the analyses of cortical processes, future studies should focus on the question which perceptive-cognitive processes form the basis of human haptic perception. In our opinion, more detailed analyses of ES could contribute essentially to the understanding of human haptic perception—maybe as much so, as the analysis of fixation pauses contributed to the understanding of visual perception.

In that regard, it would also be interesting to analyze whether ES occur when additional visual information is present during haptic exploration. During all present experiments the participants' eyes were closed. Futures studies should examine if a functional correspondence exists between fixation pauses of the eyes and ES of the haptic system. Additionally, the occurrence of ES in congenitally blind participants should be tested. Although Braille reading studies have shown that the fingers regularly stop during the reading process (Millar, 1987; Appelle, 1991; Davidson et al., 1992), it is not yet known, whether ES occur in congenitally blind persons during the exploration of objects and surfaces as well.

## Author contributions

Martin Grunwald experimental design, data analysis, wrote the paper. Manivannan Muniyandi, Stephanie Mueller, Frank Krause data analysis, discussion the results, wrote the paper. Hyun Kim and Jung Kim experimental hardware and software design. Mandayam A. Srinivasan experimental design, discussion.

### Conflict of interest statement

The authors declare that the research was conducted in the absence of any commercial or financial relationships that could be construed as a potential conflict of interest.
